# A Novel Approach for Non-Invasive Lung Imaging and Targeting Lung Immune Cells

**DOI:** 10.3390/ijms21051613

**Published:** 2020-02-27

**Authors:** Amlan Chakraborty, Simon G. Royce, Cordelia Selomulya, Magdalena Plebanski

**Affiliations:** 1 Department of Chemical Engineering, Monash University, Melbourne, VIC 3800, Australia; amlan.chakraborty@monash.edu; 2 Department of Pharmacology, Monash University, Clayton, VIC 3800, Australia; simon.royce@monash.edu; 3 School of Chemical Engineering, UNSW, Sydney, NSW 2052, Australia; 4 School of Health and Biomedical Sciences, RMIT University, Melbourne, VIC 3083, Australia

**Keywords:** Magnetic Resonance Imaging, nanoparticles, cellular uptake, pulmonary delivery, theranostic

## Abstract

Despite developments in pulmonary radiotherapy, radiation-induced lung toxicity remains a problem. More sensitive lung imaging able to increase the accuracy of diagnosis and radiotherapy may help reduce this problem. Super-paramagnetic iron oxide nanoparticles are used in imaging, but without further modification can cause unwanted toxicity and inflammation. Complex carbohydrate and polymer-based coatings have been used, but simpler compounds may provide additional benefits. Herein, we designed and generated super-paramagnetic iron oxide nanoparticles coated with the neutral natural dietary amino acid glycine (GSPIONs), to support non-invasive lung imaging and determined particle biodistribution, as well as understanding the impact of the interaction of these nanoparticles with lung immune cells. These GSPIONs were characterized to be crystalline, colloidally stable, with a size of 12 ± 5 nm and a hydrodynamic diameter of 84.19 ± 18 nm. Carbon, Hydrogen, Nitrogen (CHN) elemental analysis estimated approximately 20.2 × 10^3^ glycine molecules present per nanoparticle. We demonstrated that it is possible to determine the biodistribution of the GSPIONs in the lung using three-dimensional (3D) ultra-short echo time magnetic resonance imaging. The GSPIONs were found to be taken up selectively by alveolar macrophages and neutrophils in the lung. In addition, the GSPIONs did not cause changes to airway resistance or induce inflammatory cytokines. Alveolar macrophages and neutrophils are critical regulators of pulmonary inflammatory diseases, including allergies, infections, asthma and chronic obstructive pulmonary disease (COPD). Therefore, pulmonary Magnetic Resonance (MR) imaging and preferential targeting of these lung resident cells by our nanoparticles offer precise imaging tools, which can be utilized to develop precision targeted radiotherapy as well as diagnostic tools for lung cancer, thereby having the potential to reduce the pulmonary complications of radiation.

## 1. Introduction

Pulmonary radiotherapy is one of the standard treatments for non-small cell lung cancer [1]. However, radiation-induced lung injury is very common with severe acute side-effects, such as radiation pneumonitis, and chronic side-effects, such as radiation-induced pulmonary fibrosis [2]. The condition is worse for patients with pulmonary diseases as it changes their pulmonary function [3] and leads to fatal or life-threatening complications [4,5]. One of the reasons for gratuitous complications with the use of pulmonary radiotherapy is the lack of high-precision radiation techniques as well as off-target effects [2]. Therefore, the unmet need is to develop precision based pulmonary imaging tools to support accurate radiotherapy without compromising on off-target effects. Pulmonary imaging using nanoparticles is one of the emerging approaches for precision-based imaging. However, there has been concern in the literature that some of them may promote lung pathology, since a number of man-made and environmental nanoparticles cause asthma exacerbations [6,7,8,9], and are associated with size-dependent cytotoxicity in mammalian cells [10,11,12,13]. Nanoparticle size is also important in terms of nanoparticle distribution in the lung [14] and plays a major role in their overall systemic clearance and biodistribution. Usually, particles with a size of less than 5 nm are cleared by the kidneys [15], while 50–100 nm particles lodge in the liver [16]. Oftentimes, bigger micron-sized particles end up in the spleen [17], and coating the particles with dextran can be used to modulate their excretion [18]. Some nanoparticles are cytotoxic [10,11,12,13], related in some cases with additional physicochemical aspects such as charge [19,20]. Targeting nanoparticles for uptake by alveolar macrophages has recently been shown to be a useful intervention to treat lung cancer [21], and given their central role in the control of many diseases of the lung, it is likely to be useful across multiple diseases. However, since uptake by alveolar macrophages of pro-inflammatory or toxic nanoparticles and microparticles, including those made from some forms of silica and asbestos, can lead to pulmonary fibrosis and neoplasia [22], it will be useful to develop nanoparticle scaffolds that do not trigger potentially damaging inflammatory pathways during particle uptake. The uptake of diverse types of nanoparticles and microparticles in the lung by a range of endocytic cell subtypes has recently been extensively reviewed [23].

The charge on the nanoparticle surface is a critical determinant of its stability in vivo; therefore, as well as size, the zeta-potential of the particles is also important [24]. For instance, the coating on the particles surface can enhance their circulation time in the body by preventing efficient uptake by antigen presenting cells [25], or precluding aggregation due to a biological fluid such as serum or mucus [26,27]. Overall, amino acid coatings can be used to improve the biocompatibility, size and biodistribution, as well as potential interaction with immune cells, making them ideal molecules for conjugating on nanoparticles for therapeutic and diagnostic applications [28,29,30]. Therefore, the coating on the particle, along with its core, will both be significant when designing particles for pulmonary delivery and imaging.

Different coatings that can help modify the biodistribution of the particles in vivo include polyethene glycol, dextran and amino acids [29], which contain methyl, amino and carboxy functional groups. The availability of two functional groups (amino and carboxy) is beneficial as one can be utilized for attachment of drugs and other molecules, while the other is accessible to the nanoparticle. For enhanced and accurate imaging, iron oxide nanoparticles have been proven to be successful in Magnetic Resonance Imaging (MRI) of tumors and soft tissues [31,32] and their biodegradable nature prevents accumulation in tissues [33]. The advantage of MRI over fluorescent probes and/or nanoparticles is the localization of the signal within tissue, MRI can image deep within the body, while fluorescence is limited in penetration by tissue scattering of the emitted light. Additionally, the fluorescent probe can be cleaved from the nanoparticle leading to a false positive signal. Biodegradable nanoparticles with super-paramagnetic properties are useful as MR contrast agents as the core of the particle is responsible for image contrast [32] with improvement in contrast when two adjacent areas have difference in signal intensity (SI). Contrast difference is required to differentiate between the normal anatomy and pathology, which enhances and consolidates our understanding of the full spectrum of these manifestations to improve pulmonary imaging. Therefore, signal intensity on an MR image is governed by many factors including the tissue characteristics, proton density and type of sequence used [34].

Despite recent advancements in MR imaging of tumors [31,32] and soft tissues such as liver [35], non-invasive lung imaging is difficult and expensive with hyperpolarized noble gases used as contrast agents [36]. However, the works by Wyszogrodzka et al. showing iron metal-organic-frameworks for intranasal drug delivery demonstrates advanced ways of MR imaging of the lungs for theranostic purposes [37,38]. Therefore, the potential to improve lung MR imaging is extensive. Pulmonary inflammation is a hallmark in many respiratory diseases in which cells such as neutrophils and alveolar macrophages are the key players in regulating inflammation. An unmet need would be in tracking functional immune regulatory lung cells, such as alveolar macrophages and neutrophils, and understanding nanoparticle biodistribution. A key problem with current lung MR imaging is the hollow nature of the lung, comprising air and negligible hydrogen protons, which are required for standard MR imaging [39]. In addition, the lung is not a static organ as it is consistently under a rhythm of inhalation-exhalation further complicating the imaging. To overcome this problem, we used a non-invasive three-dimensional (3D) ultra-short echo time (UTE) MR imaging sequence in order to nullify respiratory and cardiac motion [40]. With near zero echo times such as ultra-short echo time sequences, have shown utility for the assessment of non-cell lung carcinoma and pulmonary edema [41]. In order to study nanoparticle biodistribution, we synthesized and delivered intranasally, super-paramagnetic iron oxide nanoparticles coated with the amino acid glycine (GSPIONs) to investigate the uptake by cells resident in the lung. Glycine is the simplest neutral amino acid; it does not induce inflammation and being small it minimizes extending ligand arms with functional groups helping control nanoparticle size. In addition, glycine has been used as excipient for GSPION delivery by preventing mucociliary clearance [30]. We demonstrate for the first time the biodistribution of GSPIONs within the lungs of mice following inhalation using a 9.4T magnet and a non-invasive 3D ultra-short echo time (UTE) MR imaging sequence [40]. Utilizing the γ-Fe_2_O_3_ core of the nanoparticles, we were able to show selective nanoparticle uptake by specific alveolar macrophages and neutrophils. We measured airway resistance and local pro-inflammatory cytokine production in the lung after GSPION administration and were able to confirm the particles were non-inflammatory. The data show a promising safety profile for nanoparticles coated in this natural amino acid and a useful platform to further explore pulmonary imaging in respiratory diseases. Better imaging, leading to more precise radiotherapy, may offer more powerful and safer treatments to patients by reducing radiation-induced complications.

## 2. Results

### 2.1. Characterization of GSPIONs for Evaluating Their Pulmonary Delivery Capability

**Figure 1:**
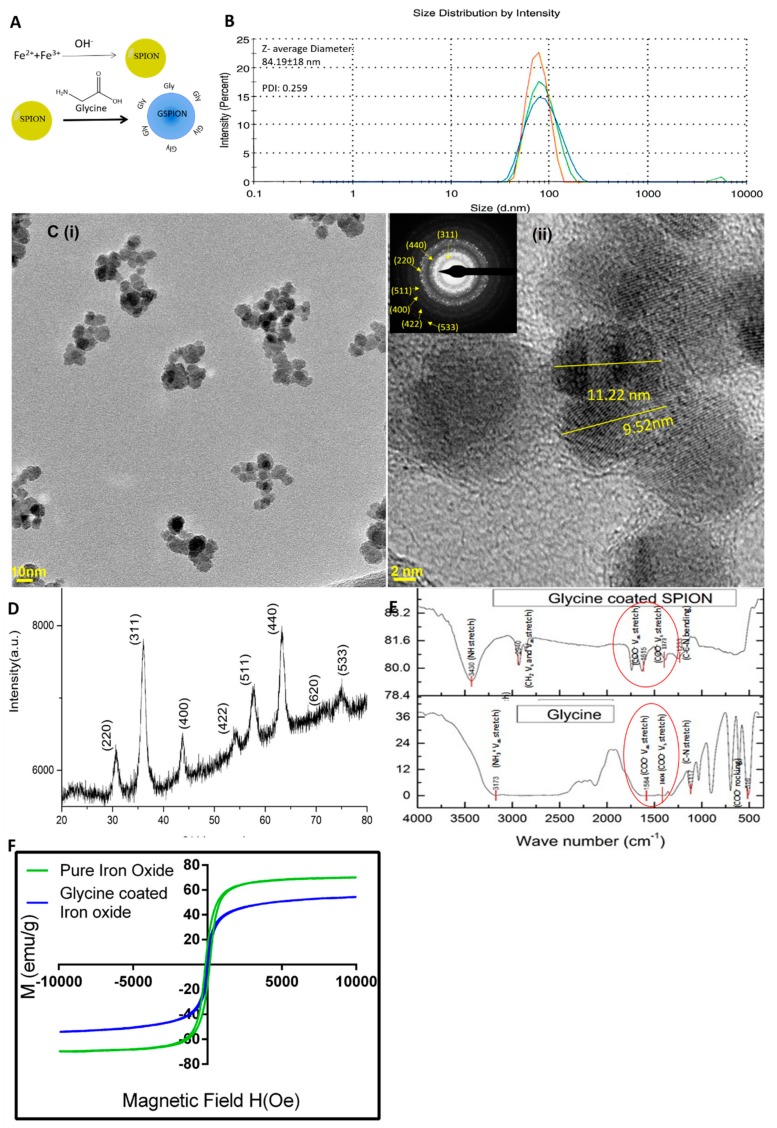
Synthesis and characterization of Glycine coated Super-Paramagnetic Iron Oxide Nanoparticles (GSPIONs). (**A**) GSPIONs are synthesized using alkaline co-precipitation followed by glycine addition in a single reaction vessel under inert atmosphere of N_2_ where glycine is chemisorbed into nanoparticle surface. (**B**) Dynamic light scattering (DLS) to determine hydrodynamic diameter of nanoparticles (intensity vs. size distribution plot) showing a mean diameter of 84.19 nm with a poly-dispersity index of 0.259. Three out of eight representative experimental data is shown. (**C**) HR-TEM images of GSPIONs (**i**) showing high dispersion and less clusters (scale bar 10 nm); (**ii**) showing cubic nanoparticles of average size 11.2 nm (scale bar 2 nm) and (**inset**) SAED pattern showing electron diffraction due to different lattice planes, demonstrating crystallinity of the GSPIONs. (**D**) XRD spectra of GSPIONs showing eight characteristic peaks and two signature peaks at (620), (533) corresponding to maghemite (γ-Fe_2_O_3_); (**E**) FTIR spectra of glycine alone (showing V_s_ and V_as_ COO^−^ bands) shown with red circles and GSPIONs with corresponding functional groups shown in red circles between wave number 450–4000 cm^−1^; (**F**) Field dependent magnetization plots of pure iron oxide nanoparticles and GSPIONs at 300 K showing superparamagnetic behavior.

Glycine coated super-paramagnetic nanoparticles (GSPIONs) were synthesized using a modified alkaline co-precipitation method in order to generate crystalline nanoparticles suitable for imaging applications (Figure 1A). The method used further enables the chemisorption of glycine onto the nanoparticle surface, as described for glycine by Barick et al. [42] and previously by our group [30]. The synthesized GSPIONs were monodisperse with a low standard deviation (Figure 1Ci). The nanoparticles size, as measured using the Quantax system, had an average size of 12 ± 5 nm (Figure 1Cii). The shape of the nanoparticles was found to be cubic and crystalline. There were eight rings present in the SAED pattern, corresponding to a crystalline structure (Figure 1Cii-inset). The SAED pattern was indexed to crystalline reflections at (220), (311), (400), (422), (511), (440), (620) and (533), which signifies the possibility of γ-Fe_2_O_3_. Due to the prolonged reaction time, the nanoparticles had a higher crystallinity and addition of glycine at a later stage aided the crystal growth, increasing the degree of crystallinity in a similar manner observed earlier [42]. In general, nanoparticles in the hydrodynamic size range of 50 nm are considered ideal for uptake by antigen presenting cells [29,43]. In addition, for pulmonary delivery, nanoparticles (< 1 µm) are needed to reach the level of the alveoli [23]. Therefore, we measured the hydrodynamic size of the GSPIONs using Dynamic Light Scatering (DLS) over eight replicates. The GSPIONs had an excellent poly-dispersity index (PDI) of 0.259, which signified a high amount of stability in suspension without clustering (Figure 1B). This can be attributed to the glycine coating on the nanoparticle surface, as SPION nanoparticles otherwise agglomerate due to electrostatic attraction. The hydrodynamic diameter of the GSPIONs was measured to be 84.19 ± 18 nm, therefore, showing useful size range for pulmonary delivery into the alveoli (Figure 1B). The PDI of the nanoparticles also explained their colloidal stability. The GSPIONs exhibit superparamagnetic behavior without magnetic hysteresis and remanence at 300K. The maximum magnetizations of GSPIONs (at an applied field of 10 KOe) was found to be 54.42 emu/g respectively (Figure 1F). 

To confirm the chemisorption of glycine on the nanoparticle surface, we used IR spectroscopy and Carbon, Hydrogen and Nitrogen (CHN) analysis. The absorption bands for glycine were found to be resolved properly as it is a zwitterionic compound. However, that of GSPIONs are broad (Figure 1E). There is a shifting of NH^3+^ V_as_ stretching in pure glycine to (NH) and CH_2_ stretching vibration at 3430 cm^−1^ and 2940 cm^−1^ respectively in GSPIONs. Stretching vibration in pure glycine at 1117 cm^−1^ shifted to a bending *C-C-N* vibration at 1233 cm^−1^ and absence of COO^−^ rocking vibration in GSPIONs. The V_s_ and V_as_ stretching bands of COO^−1^ group of glycine are shifted from 1404 to 1373 cm^−1^ and 1584 to 1615 cm^−1^ along with a wave number separation of Δ=242 cm^−1^ between V_s_ COO^−^ and V_as_ COO^−^. This type of covalent bonding suggests that glycine is chemisorbed onto the surface of the nanoparticles through carboxylate groups with freely exposed amine (-NH_2_) groups. CHN analysis quantifies the elemental presence of the elements carbon (C), hydrogen (H) and nitrogen (N) in a sample. Therefore, we utilized this method in order to find the presence as well as quantify the number of glycine molecules chemisorbed per nanoparticle on to the surface of GSPIONs (Table 1). Glycine has two carbons, five hydrogens and one nitrogen. Hence, nitrogen was used as the element to evaluate the number of glycines conjugated. The particles show themselves as a cubic lattice and their diameter varies from 4.5 to 12, which we had seen in High Resolution- Transmission Electron Microscopy (HR-TEM) micrographs using Quantax analysis. Therefore, glycine content is estimated by a function of the nitrogen content and size of the particles as proposed below, modified from the formula used by Barick et al. [42] and calculated using Wolfram Alpha^®^ (shown in Supplementary Figure S1):

Number of glycine molecules per nanoparticle = $\int_{a=4.5}^{12}6a^{2}\int_{N=18.39}^{19.16}\frac{N}{14}dNda$

where a is the side length of the cube where the side length ranges from 4.5 nm to 12 nm and N is the percentage of nitrogen, resulting in an estimated average 20.2 x10^3^ glycine molecules conjugated in each particle.

### 2.2. GSPIONs Are Distributed Throughout the Lung and Show T2* Relaxivity in Lung

**Figure 2:**
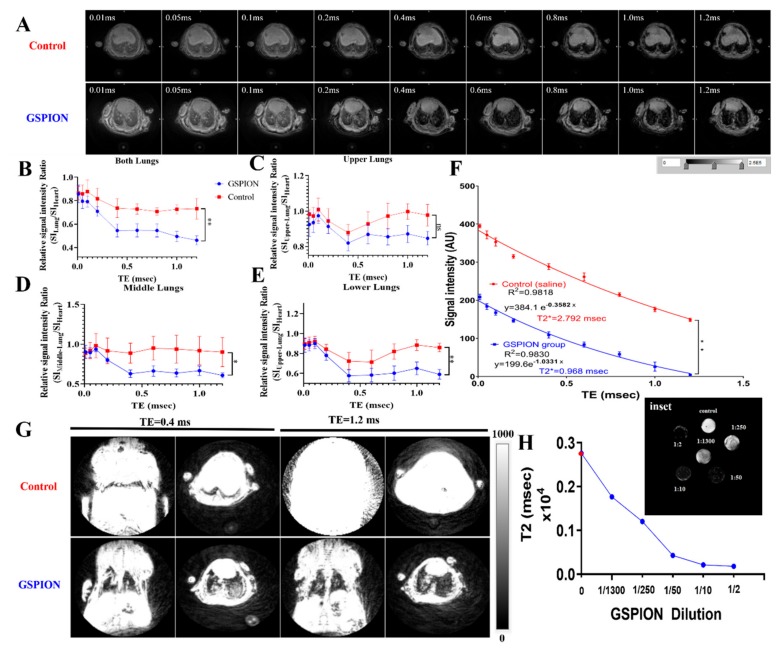
Non-invasive three-dimensional (3D) Ultra-short Echo Time (UTE) MR imaging of the lung for biodistribution. All MR images are at slice 71 for group GSPIONs (represented in blue) and control (saline, represented in red). (**A**). GSPION vs. Control (0.9% saline) sensitized mice imaged at different TEs between 0.01 to 1.2 msec. (**B**). Relative signal intensity ratio (SI_Lung_/SI_Heart_) for the whole lung and heart was calculated using ROI, shown in supplementary Figure 2, to show the difference in between the GSPIONs vs. control (saline) administered groups. The relative signal intensity ratio for GSPIONs group was lower in comparison to saline. (**C**)**.** Signal to noise ratio of the upper lung region for GSPION vs. control (saline) groups. (**D**). Signal to noise ratio of the middle lung region for GSPION vs. control (saline) groups. (**E**). Signal to noise ratio of the lower lung region for GSPION vs. control (saline) groups. (**F**). Signal Intensity decay curves of GSPION treated group in comparison to control (saline). Mono-exponential fitting equation and R^2^ values are indicated for five mice in GSPION group and three mice in control group. The SIs were measured for all TEs ranging from 0.01 to 1.2 msec, which demonstrates an exponential decay of the order S = S0e-TE/T2*. Based on the equation, T2* was calculated for GSPION and control (saline) group(**G**). 3D UTE volumetric rendering of GSPION sensitized and control mice at ultra-short TE 0.4 msec and conventional TE 1.2 msec. The lung in GSPION groups are visible from the signal contrast by the GSPIONs, while the saline (control) group lacks contrast from cardiac muscle and other tissues. The lungs are prominent in the GSPION treated group, while in the Saline treated group, we found them barely visible due to attenuation of signal by the accumulation of the GSPIONs, therefore showing their distribution in the lung. (**H**). The T2 (msec) of phantoms is represented as a function of the GSPION dilutions with control (in red) resembling 0.9% saline in agarose. (**inset**) T2 (msec) of different GSPION dilutions (resembling concentrations) showing relaxivity and darkening due to GSPIONs presence. For this experiment, N = 5 mice/group for GSPIONs and N = 3 mice/group for control (saline) was used, and Signal to noise ratio was represented as Mean ± SD. To assess the difference between the relative signal intensity ratio of the two groups across different TE’s, an unpaired T test with Welch’s correction (not assuming equal SD) was used to determine the significance. **p < 0.005, *p < 0.05, ns; non-significant.

Typical ultra-short echo time (UTE) images of the lungs in mice instilled with saline or GSPIONs at nine different echo times (TEs) ranging from ultrashort (0.01 msec) to conventional (1.2 msec) are shown in Figure 2A. The images were “motion insensitive” although no gating was implemented, similar to Takahashi et al. [40]. Signal intensity (SI) was consistent within the cardiac muscle that we used as an internal imaging control and was used to normalize for every TE across each mouse. We calculated the relative signal intensity ratio by dividing the SI in the lung over the SI in the heart (SI_Lung_/SI_Heart_). The signal to noise ratio of the overall lung (SI_Lung_/SI_Heart_) for GSPION sensitized mice was significantly lower [44] in comparison to saline-treated mice (Figure 2B) for any TE. In general, the GSPIONs showed a reduction in signal intensity consistent with the effects of iron oxide nanoparticles visible across the phantoms (agarose + GSPIONs) at different concentrations scanned (shown in Figure 2H) in comparison to control (agarose + saline). For determining the relative signal intensity ratio in lung parenchyma, the lung was segmented into upper, middle and lower regions. Interestingly, the relative signal intensity ratio for control (saline-treated) mice was always higher in all the lung segments (upper, middle and lower) than GSPION sensitized mice (Figure 2C–E). However, only the middle (SI_Middle_/SI_Heart_) and lower (SI_Lower_/SI_Heart_) segments had a significant difference in the relative signal intensity ratio between the two groups. The difference in relative signal intensity ratio in between the GSPION and saline (control) groups across the middle and lower regions indicated a higher concentration of particles in the lower alveolar regions, owing to a higher decrease in the contrast between the lung and the cardiac muscle. The SIs measured at a range of TEs from 0.01 to 1.2 msec demonstrated excellent mono-exponential fitting for the GSPION group (R^2^ = 0.9830) and control (saline) group (R^2^ = 0.9818). The Region of Interest (ROI) for the measurements is represented in supplementary information Figure 3. T2* for saline (control) was calculated as 2.792 msec, while that of GSPIONs group was 0.968 msec (Figure 2F). The T2* (msec) of the lung in the GSPION group was significantly lower (*p* < 0.005) than the control (saline) group. Using the 3D volumetric rendering algorithm of ImageJ, we used the information in the 3D voxels to join and create a 3D surface model (Figure 2G). The 3D rendering illustrated the difference between the GSPION sensitized groups and that of the saline (control) group. There was a significant difference in contrast (visible in Figure 2G) between the lungs and the cardiac muscle including the surrounding tissue visible in the GSPION group only. However, the 3D rendering demonstrated no such contrast difference in the saline (control) treated group. We investigated the lung surface across TE (0.01 and 1.2 msec) for both the groups. The lung of the saline group was prominently visible (shown in red), while that of the GSPION group (shown in blue) was barely visible accounting to the decrease in relative signal intensity ratio and T2* due to accumulation of GSPSIONs. The GSPIONs were evenly distributed between the left and right lobe of the lung, therefore, showing consistency of administration of the GSPIONs across different mice. The T2 distribution across different dilutions of the GSPIONs in agar (phantoms) resembling the concentration of GSPIONs and saline (control) are shown in Figure 2H. The decaying intensity resembles the change in contrast across the tissue in the two groups shown in Figure 2A. The dark patches in the lung parenchyma are consistent with the biodistribution of GSPIONs following inhalation (Figure 2A,G) but not visible in saline controls.

### 2.3. Selective Localization of GSPIONs in the Lung Within Alveolar Macrophages and Neutrophils

**Figure 3:**
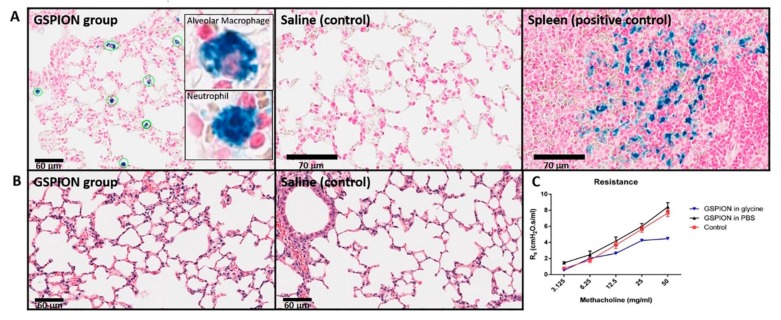
The therapeutic ability of GSPIONS to alleviate lung resistance was not directly tested, although their localization into immune cells suggests this potential. (**A**). Perls’ Prussian blue counter stained with neutral red to determine uptake by immune cells in the lung for GSPIONs and saline group and spleen showing positive stain due to hemosiderin. (**inset**) GSPIONs taken up by alveolar macrophages and neutrophils confirmed by nucleus visible after Perls’ Prussian blue counter stained with neutral red. (**B**). Hematoxylin and Eosin stain showing lung parenchyma in both GSPION and saline (control) group. (**C**). Lung resistance measured by invasive plethysmography in GSPIONs suspended in glycine and PBS in comparison to control. No significant difference was observed in between control and GSPION groups.

GSPIONs were delivered intranasally by suspending either in Phosphate-buffered Saline (PBS) (as recovered from reaction) or in glycine and compared to saline (vehicle control) followed by invasive plethysmography, H&E and Perls’ Prussian blue staining (Figure 3A). GSPIONs were taken up by different immune cells as they stained blue due to Perls’ Prussian blue stain (Figure 3A). The cells are circled (in green) taking up the particles and are absent in the saline (control) group, which is attributed to the absence of particles. Specifically, alveolar macrophages and neutrophils were found to take up the GSPIONs (Figure 3A-inset) and was confirmed by Perls’ Prussian blue stain. Hemosiderin is deposited in spleen as a result of rupturing of red blood cells, releasing iron. Therefore, we utilized the spleen as a control for positive staining as it is similar to the iron crystal structure of our GSPIONs. The GSPIONs were not found in the heart or liver (shown in Supplementary Information Figure 3), indicating their localization in the lung. The lung parenchyma showed no signs of damage when compared to saline (Figure 3B). By invasive plethysmography, lung resistance was measured for all the groups. It was found that GSPIONs suspended in glycine showed lower resistance in comparison to GSPIONs suspended in PBS (Figure 3C). However, the resistance of GSPIONs in PBS was comparable to saline (control).

### 2.4. GSPIONs Do Not Induce Pro-Inflammatory Cytokines

**Figure 4:**
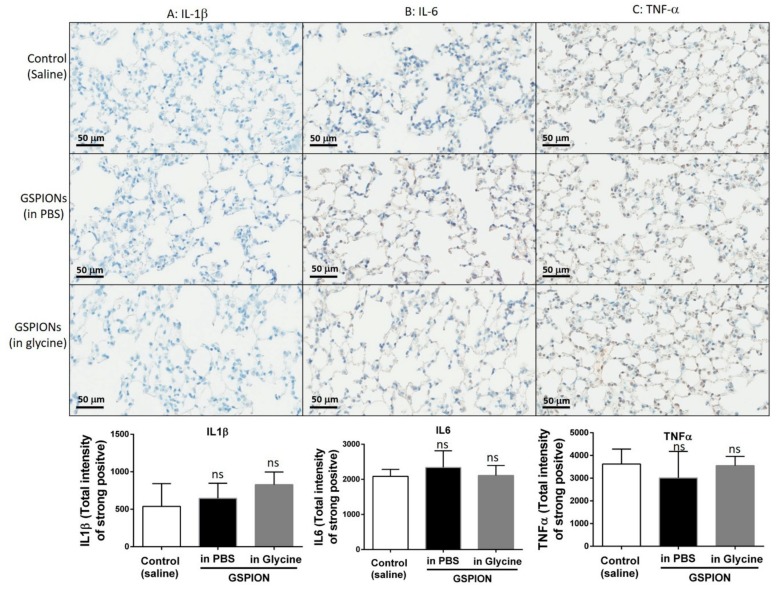
GSPIONs do not increase expression of pro-inflammatory cytokines in lung parenchyma. In comparison to control (saline), expression of pro-inflammatory cytokines, (**A**). IL-1β, (**B**). IL-6 and (**C**). TNF; was unchanged along with negligible damage in the lung parenchyma. Scale bars represent 50 µm. N = 6 mice/group, Mean ± SEM. A one-way ANOVA was used to determine the significance in between different groups. Each group was quantified for strong positive expression by analyzing 10 sections/lung/group. *ns, non-significant*.

Expression of pro-inflammatory cytokines IL-1β, IL-6, Tumor Necrosis Factor (TNF) was investigated upon administration of GSPIONs suspended in PBS or glycine and compared to control (vehicle) into the lung (Figure 4). In each section/mice/group, 10 regions were selected using the positive pixel count algorithm, avoiding muscles to quantify the amount of positive stain for each of these cytokines as heat maps to represent the different intensity of expression. The background was subtracted from the positive pixel intensity and recorded as the intensity of a strong positive. Expression of pro-inflammatory cytokines in both GSPIONs suspended in PBS or glycine did not elicit an immune response resulting in a higher expression compared to saline. Parallel studies with LPS by contrast showed clear and significant cytokine upregulation (Supplementary Information Figure 4). The background expression of IL-1β was comparatively lower to that of IL-6 and TNF in all groups. The unchanged expression of pro-inflammatory cytokines clearly indicates that these GSPIONs, unlike naked iron oxide nanoparticles, do not initiate an inflammatory reaction [45,46]. Therefore, these GSPIONs offer a potentially useful new platform for the development clinical applications such as in diagnostics and therapeutics.

## 3. Discussion

Properties of nanoparticles such as size, shape and charge are very important for interaction with immune cells. Regarding nanoparticle size in pulmonary delivery, dense microparticles (> 5 µm) are deposited in the trachea, while particles between 1–5 µm are phagocytosed [47]; nanoparticles with a size less than 1 µm end up in the alveoli [48]. These nanoparticles are then taken up by highly endocytic cells that are present in the lung, such as alveolar macrophages and neutrophils. These in turn are changed in number under diverse environmental challenges, such as infections or pollution, or due to the presence of cancer. Our nanoparticles have a hydrodynamic size of approximately 84.19 ± 18 nm, which is ideal for reaching the lung alveoli during pulmonary delivery and the MRI as well as histology data suggest the particles reach the lower levels of the lung within the alveoli spaces. Very recently, it has been demonstrated that macrophages (especially alveolar macrophages) take up iron oxide nanoparticles with an average size range (measured by TEM) of 10 nm [49,50]. Our GSPIONs are of the size 12 ± 5 nm as measured by TEM, and we show that they are preferentially taken up by alveolar macrophages and neutrophils. The assembly of nanoparticles observed on our DLS measurement of the hydrodynamic size can be attributed to the presence of sodium chloride during synthesis and washing, increasing the solvation layer around the particles. The transition from ferro or ferrimagnetic behavior to superparamagnetic behavior at room temperature was attributed to glycine chemisorption. The room temperature magnetization of GSPIONs was reduced to 88% respectively of the bulk Fe_2_O_3_. Hence, the GSPIONs demonstrate super-paramagnetic behavior and we refer to them as GSPIONs. Furthermore, the coating with glycine was advantageous as it prevented the agglomeration, unlike naked iron oxide nanoparticles, while at the same time being a useful inert coating for use in patients with diabetes [29]. Although a very minute amount of glycine is being coated on to the nanoparticle, yet it has several advantages for future clinical use [30]. Glycine is known to be sweet and improves insulin sensitivity along with a reduction of symptoms induced by hyperglycemia [51,52], albeit at higher concentrations than what we coated our nanoparticles. Our GSPIONs do not escalate inflammation, unlike other naked nanoparticles, in a size range of 30–300 nm [53]. In addition, nanoparticles within 5 nm often accumulate in the nucleus of cells, while that of 15–40 nm end up in the cytoplasm [29]. In addition to the ideal size for cellular uptake and pulmonary delivery, magnetic nanoparticles have the capability of magnetic hyperthermia whereby the nanoparticles are used for the controlled generation of heat to kill cancer cells while reducing damage to surrounding tissue [54]. Our GSPIONs being superparamagnetic share similar properties to those illustrated by Barick et al. [42]. Therefore, GSPIONs may also provide a base to be functionalized further for magnetic hyperthermia therapy applications in the directed killing of malignant tumor cells without damaging local tissue. Overall, the GSPIONs are ideal for further development of therapeutic applications in the lung, aiming to reach deep alveoli, without by themselves inducing inflammation [30]. Although studying diverse types of coating is beyond the present study, we hope these results will encourage further investigation into this approach.

Nanoparticles are not only useful for tumor ablation, but are also useful as imaging agents, although there is a significant gap in knowledge regarding pulmonary magnetic resonance imaging. Magnetic resonance imaging of lung is limited due to the low density of H-protons in air and a relative lack of water. The problem can be avoided with 3D ultra-short echo time MR imaging as has been used multiple times very recently [37,38,40]. 3D UTE MRI adequately generated signal from lung parenchyma that was decreased by the presence of GSPION’s (the heart being an internal endogenous negative control) and showed that GSPIONs could be delivered and used as novel diagnostic agents due to their distribution into the alveolar spaces along with stratification within lung lobes. Some studies have reported reduced relaxivity of superparamagnetic nanoparticles upon cellular uptake [55,56], but our functionalized glycine coated GSPIONs continue to reduce signal intensity 24 h after administration when histology results suggest they are preferentially taken up by phagocytic cells. The results thus far demonstrate that this non-invasive technique can determine GSPION distribution in the healthy lung. The results would be useful for diagnosis of respiratory diseases where emphysema and edema are observed. In addition, our results demonstrate, for the first time for glycine coated biodegradable nanoparticles, a volumetric differentiation of nanoparticle distribution when standardized to cardiac tissue, which can segregate their biodistribution depending on the signal intensity observed in the lung slices. The imaging features reported contribute to the overarching aim to enhance targeted radiotherapy and would help in developing more precise approaches. Future studies (beyond the scope of this work) may test the potential range of therapeutic and enhanced imaging approaches against diverse lung diseases including cancer, asthma and COPD, utilizing several time points as the advantage of MRI promotes longitudinal study. For example, this differentiation may be useful in future applications for determining drug delivery, and for identifying lung-lobe specific targets in diseases such as asthma (where the upper airways and lobes are targeted) or COPD and fibrosis (where the lower lobes are affected) [14,47]. Therefore, it indicates the necessity to directly address such issues in the above-mentioned disease, as these may also differ depending on the disease studied.

Uptake of compounds by immune cells is difficult to study. Our results using GSPIONs show them to be localized in vivo within alveolar macrophages and neutrophils. It clearly indicates that cells responsible for pro-inflammatory cytokines secretion leading to pulmonary inflammation could be passively targeted using these GSPIONs. In addition, macrophages have elevated plasticity, and can be useful as therapeutic targets. For example, in a tumor microenvironment, macrophages play a pro-tumoral role and are classified as tumor associated macrophages [57], which are directly associated with cancer [23]. Tumor associated macrophages [57] in the resting phase secrete immune-suppressive cytokine IL-10, while during activation by lipopolysaccharide (LPS), they secrete proinflammatory cytokines IL-1β, IL-6 and TNF. Successful interaction of our GSPIONs with alveolar macrophages is a suggestive indication that GSPION uptake by TAMs may also be developed as a tool to help inhibit malignant cell growth. This potential is supported by recent findings in lung adenocarcinoma, where uptake by alveolar macrophages of apoptotic drugs resulted in alveolar macrophage depletion leading to tumor reduction [21]. In other diseases such as COPD, characterized by increased alveolar macrophages leading to epithelial thickening and emphysema, the GSPIONs may be similarly useful in attenuating inflammatory conditions. The uptake of GSPIONs by lung resident cells enables us to perceive future-targeted therapeutics. Moreover, the results contribute to the development of targeted radiotherapy, which may involve lung resident cells. The negligible resistance of GSPIONs, which is similar to control (saline), may enable the use of these GSPIONs as immunomodulators or clinical use in contrast to other nanoparticles. The unchanged pro-inflammatory cytokines and preferential uptake by neutrophils and alveolar macrophages points out their significant use in therapies for respiratory diseases such as COPD and acute respiratory distress syndrome (ARDS). In regard to ARDS, an increased neutrophil inflammation is observed due to IL-33 being secreted by the neutrophils themselves and augmenting it by alveolar macrophages [23]. Although the GSPIONs were not designed for active targeting of neutrophils and alveolar macrophages, we do observe their preferential uptake by neutrophils as well as macrophages. This could be due to multiple mechanisms, including size and surface charge of the particles, which is common with other types of nanoparticles used in the lung [14,29,43,58], as well as more speculatively, potentially the presence of a non-neuronal glycine receptor (GlyR) on these specific cell types (supplementary information Figure 5 and refer to Eynden et al. [59]). The presence of GlyR may have contributed to the observed preferential uptake in these lung resident cell types, but this is an area for future investigation and beyond the scope of this paper. This indicates an additional potential use of these nanoparticles, for example co-delivering apoptotic drugs as discussed above. Therefore, for therapeutic and diagnostic interventions, GSPIONs are a new potentially useful candidate for use as a pulmonary delivery agent passively targeting a cohort of immune cells relevant to multiple diseases.

## 4. Materials and Methods

### 4.1. Synthesis and Characterization of GSPIONs

GSPIONs were synthesized using a modified alkaline co-precipitation method [30,42] using FeCl_3_ (Sigma-Aldrich Pty. Ltd., 44944) and FeCl_2_ (Sigma-Aldrich Pty. Ltd., 220299) in a 2:1 ratio and 1.5 M NaCl (Supelco, 106404). The salts were mixed at a final volume of 80 mL of H_2_O and stirred at 1000 RPM for 30 min, under nitrogen reflux at a temperature of 70 °C followed by addition of NH_4_OH (25%, 30 mL) (Sigma-Aldrich Pty. Ltd., North Ryde, NSW, Australia, 17093-1L). Glycine (0.3 g/mL, 4 mL) (Sigma-Aldrich Pty. Ltd., G7126) was added and the temperature was increased to 90 °C for an hour. The nanoparticles formed were separated using a magnet and washed several times with MilliQ water before centrifuging at 9000 RPM for 7 min to precipitate larger micro-sized particles. After collection, the nanoparticles were sonicated using a probe sonicator (130 W, 20 KHz, Sonics and Materials Inc., Newton, CT, USA, UP-VCX130PB) at 90% amplitude for 5 min to disperse any clusters. After dispersion, the hydrodynamic size (in terms of intensity vs cumulant diameter distribution) of the particles were measured using a Malvern zeta-sizer Nano (Nano ZS, Malvern Instruments, ATA Scientific Pty. Ltd., Taren Point, NSW, Australia) following the dynamic light scattering method. This was followed by measuring the actual size of the nanoparticles using HR-TEM. The particles were suspended on a copper grid and images were acquired using FEI Tecnai G2 F20 S-TWIN FEGTEM (Monash Centre for Electron Microscopy, Clayton) connected to a wide angle Orius SCD200D CCD camera and size measured using a Quantax analysis system. We also measured the selected area electron diffraction (SAED) pattern in order to understand whether the nanoparticles are amorphous or crystalline.

### 4.2. Study Design and Animal Ethics

**Figure 5:**
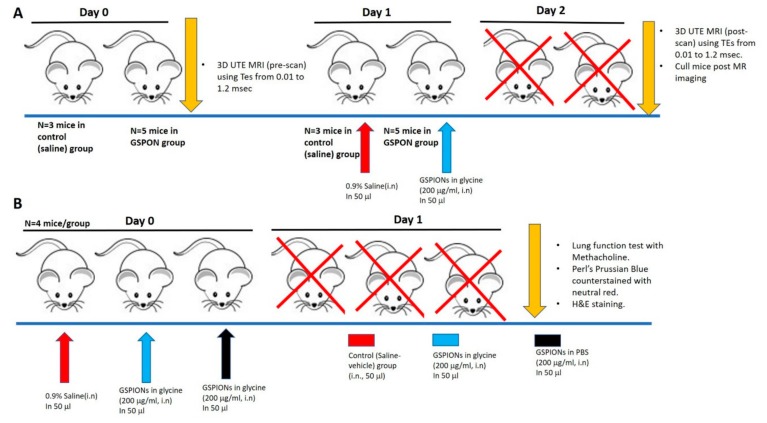
Study designs of experiments. (**A**). Study design for MR imaging experiment. Two groups are represented-GSPIONS (in blue) and control (saline-in red). Mice were pre-scanned for TEs ranging from 0.01 to 1.2 msec. The following day mice were sensitized with either GSPIONs (200 µg/mL) suspended in PBS or with control (saline). After 24 h of sensitization, mice were subjected to 3D UTE MR imaging for TEs ranging from 0.01 to 1.2 msec and culled post scanning. N = 5 in GSPION group, N = 3 in control (saline) group. (**B**). To perform acute hyperresponsiveness (AHR) and nanoparticle uptake by immune cells three groups with N = 4 mice/group were used. Mice in different groups were sensitized with GSPIONs (200 µg/mL) suspended in PBS or 100 mM glycine and 0.9% (*w/v*) saline was used as control. After 24 h of sensitization, mice were subjected to lung function test (for measurement of AHR), culled (shown by red cross) and the lungs were processed routinely with 10% neutral buffered formalin for sectioning followed by histochemical staining. Perls’ Prussian blue counter stained with neutral red to stain for GSPIONs and Hematoxylin and Eosin staining for lung architecture was performed on lung sections from mice of all the different groups.

Mice were anaesthetized using 1–2% isoflurane (IsoFlo^®^, Abbott Laboratories, North Chicago, IL, USA) along with 100% oxygen; and either GSPIONs (200 µg/mL) in PBS or control (0.9% saline, vehicle only) were intranasally administered at a volume of 50 µl distributed in both the nares. All GSPIONs are a representative batch out of 10 different batches of synthesized particles exhibiting similar particle characterization as reported in Figure 1. MR imaging was performed prior to sensitization and 24 h after sensitization. For the GSPION sensitized group (*n* = 5 mice) and control (vehicle:0.9% saline, *n* = 3 mice) mice were analyzed. The experimental design is demonstrated in Figure 5A. In another experiment, lung function (airway hyperresponsiveness-increased airways sensitivity, AHR) was measured for GSPIONs suspended in PBS, GSPIONs suspended in 100 mM glycine and control (0.9% saline, vehicle only) as demonstrated in Figure 5B. After measurement of AHR, mice were culled, and lungs were subjected to Perls’ Prussian blue staining to locate the GSPIONs. Lung architecture was also evaluated by Hematoxylin and Eosin (H&E) staining. Other organs, such as liver, heart and spleen, were checked for the presence of GSPIONs. Mice were distributed into two groups, GSPION sensitized (five mice) and saline (vehicle only)-(three mice) for lung MRI experiment as demonstrated in Figure 5A and Section 2.2. For AHR, nanoparticle uptake, histology and immunohistochemistry was checked; mice were distributed into three groups GSPIONs in PBS (four mice), GSPIONs in 100 mM glycine (four mice) and control (0.9% saline, 4 mice). The study therefore both overlaps and provides strength to the data that show that different methods in different animals give the same answer.

For all experiments, BALB/c female mice (6–8 weeks old) were maintained in the animal facility (MARP) at 22–26 °C, 55–75% humidity and a 12/12-h dark/light cycle with food and water *ad libitum*. All animal work was conducted by the approval of Monash University Animal Ethics Committee under ethics No. MARP/2017/117 in compliance with the guidelines of the National Health and Medical Research Council (NHMRC) of Australia.

### 4.3. Lung Function Measurement Upon GSPION Sensitization

Mice were anaesthetized with ketamine (100 mg/kg body weight) and xylazine (10 mg/kg body weight) by i.p. administration. The mice were then subjected to tracheostomy procedure, where a small incision was made in the trachea after a blunt dissection. Through the trachea, a 22G steel cannula connected to a tubing was inserted and sutured to support it in place. The tube was then connected to a plethysmography machine DSI Buxco - FinePointe RC system (Biosystem XA version 2.7.9, Buxco Electronics, Troy, NY, USA), ventilating the lung at normal breathing rate. AHR measurements were performed by the machine in response to increasing (doubling) dose of methacholine (3.125, 6.25, 12.5, 25, 50 mg/mL) (Mch, Acetyl-β-methacholine chloride ≥ 98% TLC, A2251) using flow and pressure transducers. Resistance (Rs) parameter data was measured (Biosystem XA version 2.7.9; Buxco Electronics) for 2 min in each mouse upon administration of PBS, which was used as background to normalize data.

### 4.4. Histology and Morphometry

Two lobes of the lung and liver, heart and spleen were immersed-fixed in 10% Neutral Buffered Formalin (NBF) followed by routine processing and embedding in paraffin. The organs were sectioned transversely at the level of the proximal intrapulmonary main axial airway in the lung and the other organs at the center level. Sections of thickness 4 µm were cut and placed on glass slides for H&E and Perls’ Prussian Blue staining.

#### 4.4.1. Nanoparticle Uptake by Lung Immune Cells

Perls’ Prussian blue along with counterstaining with nuclear fast red was used to stain the GSPIONs for identifying uptake by lung immune cells. Slides were first deparaffinized and brought to water by repeated washes with xylene, 100% alcohol, 70% alcohol and water. The first step involves the production of Prussian blue color due to the presence of γ-Fe_2_O_3_. Slides were immersed in 2% HCl and 2% potassium ferrocyanide for 20 min followed by washing in distilled water. The slides were then counterstained with nuclear fast red for 10 min followed by washing under tap water. The slides were dehydrated, cleared, mounted and imaged in an Olympus BX50 microscope. The images obtained were analyzed using Aperio Imagescope (Leica Biosystems, Nusloch, Germany). Spleen tissue from both control (saline) and GSPION treated mice were stained with Perls’ Prussian blue followed by nuclear fast red to be considered as a positive stain. There was no difference in staining of the spleen, between the saline (control) versus the GSPION treated group, hence, the Perls’ Prussian blue showed a true stain.

#### 4.4.2. H&E Staining for Tissue Morphometry

To evaluate the morphology of tissues upon subjecting the GPSIONs, the sections were deparaffinized and brought to water in the same way. The first step involved the staining of the sections with Lillie Mayer alum hematoxylin for 4 min, followed by washing under running tap water. After washing, the sections were distained in 0.3% acid alcohol and washed under running tap water. The sections were washed in Scott’s tap water followed by several rinses of the section in tap water. The final staining step involved eosin staining for 2 min followed by dehydrating the sections. Finally, the sections were cleared, mounted and scanned. The images obtained were analyzed using Aperio Imagescope (Leica Biosystems).

### 4.5. Non-Invasive Lung 3D Ultra-Short Echo Time Magnetic Resonance Imaging (MRI)

Mice were intranasally given either GSPION or control (0.9% saline, vehicle only). Magnetic Resonance Imaging (MRI) imaging was performed prior to sensitization and 24 h after sensitization as illustrated in the study design (Figure 5A). For the GSPION sensitized group (*n* = 5) and control (vehicle: 0.9% saline) (*n* = 3) mice were analyzed. For MR imaging, mice were anaesthetized with isoflurane (IsoFlo^®^, Abbott Laboratories, North Chicago, IL) in 100% oxygen. A 9.4T Agilent MRI magnet running Bruker imaging hardware was used to obtain all MRI images. Low-resolution multi-slice images were acquired of the lungs in both the transverse and coronal planes using a fast spin echo sequence. Based on the results, a volume of interest (VOI) region encompassing the thorax from the trachea to the diaphragm was positioned. On this region, a non-gated 3D radial UTE sequence [40] was performed repeatedly with various TEs (0.01, 0.05, 0.1, 0.2, 0.4, 0.6, 0.8, 1.0, 1.2 msec) in a fixed scale of receiver gain. The total number of projections was 61,214. The other imaging parameters were: 4.0 msec repetition time (TR), 50° flip angle, 35^3^ mm^3^ fields of view (FOV), 144^3^ matrix (isotropic), 100,000 Hz spectral bandwidth and a volume coil of 35 mm. Respiration of mice was maintained between 35–45 bpm by controlling isoflurane between 0.5–3%. The scan time for each mouse was 4 min 4 sec 856 msec.

#### Magnetic Resonance Image Processing and Quantitative Analysis

To measure signal intensity (SI), the lung was segmented for analysis using an open source image processing software (Mango v4.1) by Research Imaging Institute UTHSCSA (Lancaster, Martinez; www.ric.uthscsa.edu/mango). To define the ROIs, pulmonary vessels and trachea were avoided. ROIs such as upper (18.9 mm^2^), middle (12.88 mm^2^) and lower (19.75 mm^2^) for both left and right lung were drawn using the ROI tool (see supplementary Figure 3). The heart (28.18 mm^2^) was used as a reference or internal standard tissue control as the GSPIONs are not systemically circulated upon intranasal administration. Therefore, we do not expect to see any change in SI for both GSPION sensitized or saline (control) groups in the heart. For each TE, a total of 140 slices were imaged from the trachea to the diaphragm. We analyzed from slice 70–80 for SI calculations, as this region displayed the full lung volume without obstruction. SI was quantitated using the Analysis/ROI statistics menu. A T2 MR calculation was performed using a package developed by Schmidt et al. [60] in each ROI in the slope of the logarithms of noise-corrected SIs versus different TEs [40]. SI of each segment was analyzed by the Z-axis profile function. The background from the pre-sensitization images was used to analyze the SI in air to provide an estimate of noise in each image [61]. To determine the relative signal intensity ratio for data representation and statistical analysis, the SI for the whole lung or lung segment was divided by the SI for the heart, which served as the internal control. The signal of the heart was normalized across all TEs for every mouse to have consistency in calculations. The relative signal intensity ratio was calculated for every TE at slice 71 laid axially for better observation. Using the 3D volumetric rendering algorithm of ImageJ (National Institute of Health) software, we used the information in the 3D voxels to join and create a 3D surface model of the lung and thorax to determine the regions of the lung where the GSPIONs have reached. A contrasting difference from cardiac muscle including the surrounding tissue to that of the lung was evaluated.

### 4.6. Immunohistochemical Analysis of Pro-Inflammatory Cytokines

Lung sections of tissues from all three groups (Figure 5) were stained immunohistochemically for pro-inflammatory cytokines IL-1β (1:1500, bs-0812R, Bioss antibodies), IL-6 (1:2500, E-AB-40021, Elabscience) and TNF (1:250, ab6671, Abcam). The cytokines were identified by using rabbit polyclonal antibodies, with kidney and heart sections for positive controls respectively (shown in Supplementary Figure 5). The primary antibodies bound to the sections were detected using anti-rabbit EnVision (K5007, Dako, Glostrup, Denmark). The chromogen DAB was used, followed by counterstaining the sections with haematoxylin. The sections were cleared, mounted and imaged using an Olympus BX50 microscope (Leica biosystem, 40× magnification). The images were analyzed using the positive pixel count algorithm of Aperio Imagescope (Leica Biosystem, Nusloch, Germany). For one section/mice/group, several regions were identified and scored subtracting the background and recording the total intensity of the strong positives. The values are recorded, and statistics were applied.

### 4.7. Statistical Analysis

GraphPad Prism v6.0 for windows (Version 6.07, La Jolla, CA, USA) was used to construct graphs and perform statistical calculations. For each experiment, data were analyzed normally and log-transformed for normality as necessary prior to analysis by independent *t*-test, or two-way ANOVA with post-hoc Tukey’s test. Differences were considered statistically significant at the said group size and p values are denoted in the figures.

## 5. Conclusions

We created GSPION nanoparticles that are preferentially taken up by neutrophils, and alveolar macrophages in the lung. The GSPIONs provided a lower contrast to noise in comparison to saline treated mice when an ultra-short-echo time MRI sequence was utilized for imaging the lung tissue. Analysis of the change in signal intensity following GSPION administration showed the GSPIONs are delivered primarily to the lower lung lobes. The GSPIONs naturally targeted critical immune cells in the lung, without provoking respiratory changes or inflammation, and supported non-invasive imaging. Preferential uptake of the GSPIONs by alveolar macrophages and neutrophils suggests their use in targeting of these cells in therapeutic interventions in diseases involving these cells, such as in cancer, COPD and fibrosis along with other diseases. Therefore, the GSPIONs can provide a platform worth exploring to enable more accurate imaging across diverse respiratory, inflammatory diseases and cancer in the lung, supporting more precise downstream radiological interventions without negotiating with off-target effects.

## Figures and Tables

**Table 1:** C, H, N analysis to determine glycine coating on nanoparticle surface.

Name	%N	%C	%H
Glycine coated SPION	19.10 19.22	33.15 33.16	6.94 6.94
Pure Glycine	18.38 18.40	32.04 31.76	6.81 6.68

## Supplementary Materials

Supplementary materials can be found at https://www.mdpi.com/1422-0067/21/5/1613/s1.

## Abbreviations

GSPIONs	Glycine coated super-paramagnetic iron oxide nanoparticles
MRI	Magnetic resonance imaging
SI	Signal intensity
UTE	Ultra-short echo time
AHR	Acute-hyperresponsiveness
