# Supplementary material for: A Novel Approach for Non-Invasive Lung Imaging and Targeting Lung Immune Cells
Source: Int J Mol Sci. 2020 Feb 27;21(5):1613. doi: 10.3390/ijms21051613 (PMC7084491; doi:10.3390/ijms21051613)
Supplement: Supplementary file 1 [file ijms-21-01613-s001.pdf]

Supplementary Information

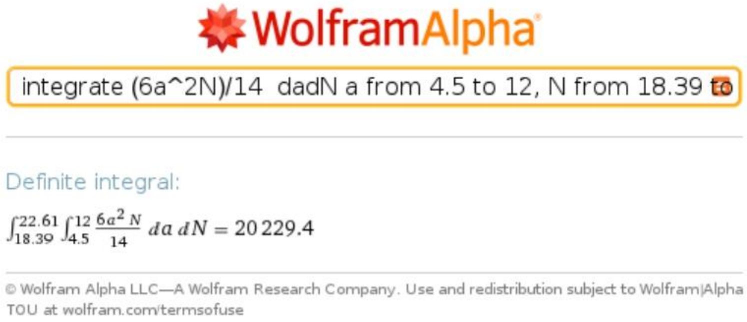

Supplementary Figure 1. Calculation of number of glycine molecules coated on nanoparticles surface using Wolfram Alpha®.

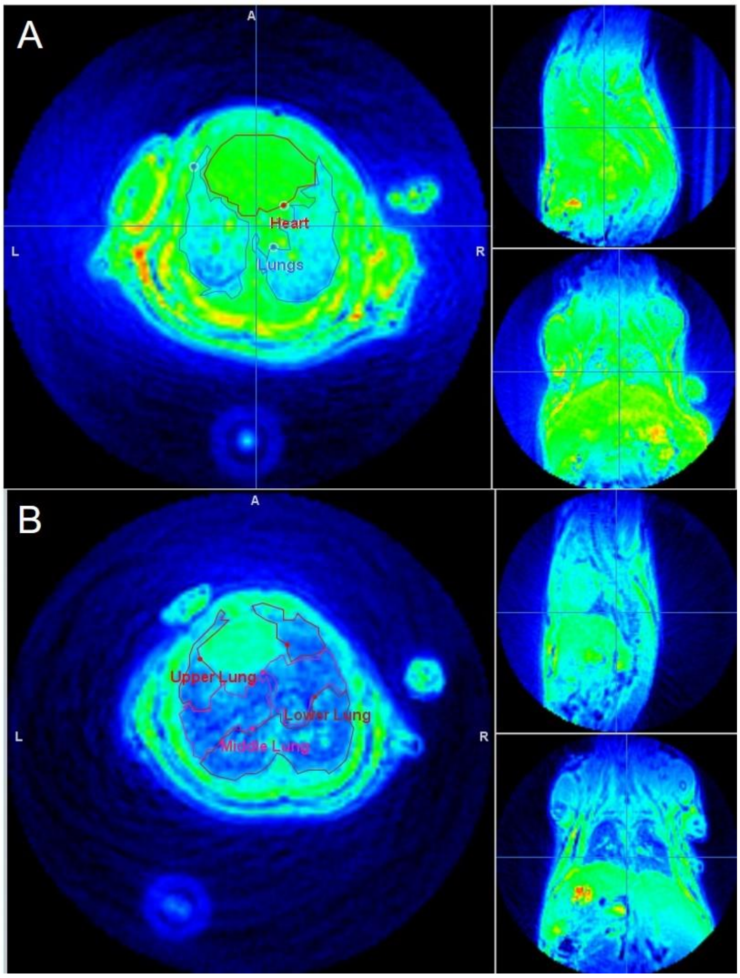

Supplementary Figure 2. ROI diagram for analysis of signal to noise ratio. (**A**). ROI of whole Lung labelled in Blue and Heart labelled in red. (**B**). ROI for upper lung in Red, lower lung in orange and middle lung in pink. In all measurements the cardiac muscle of the heart was considered as the internal standard control.

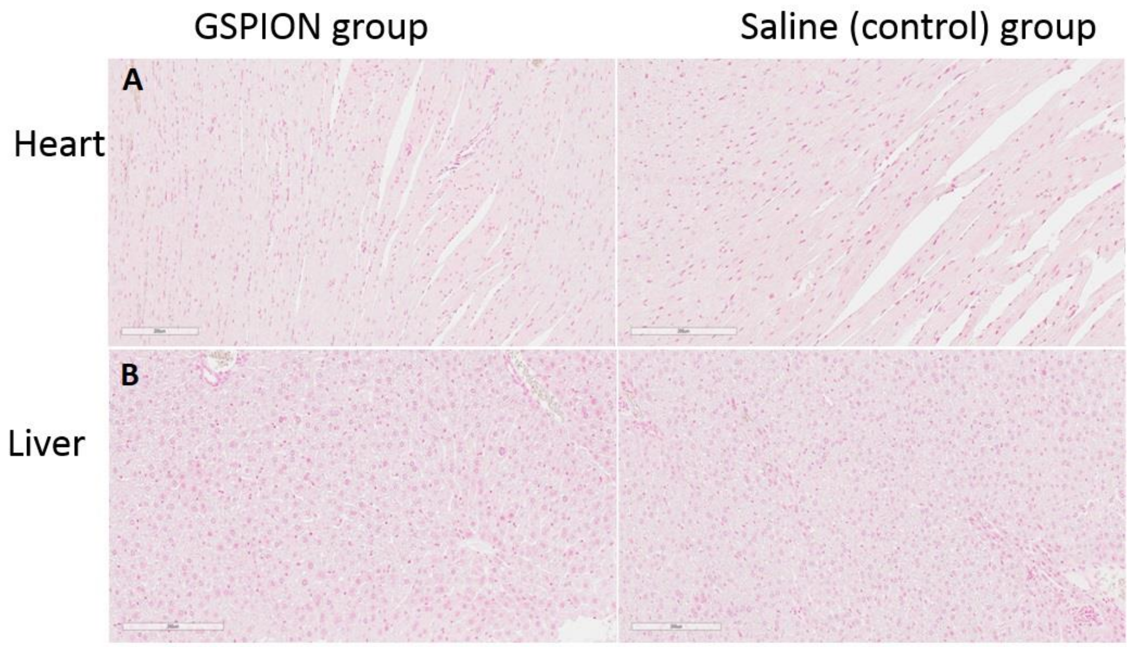

Supplementary Figure 3. Perl's Prussian blue counterstained with nuclear fast red showing absence of GSPIONs in both GSPION sensitized and saline (control) group in (**A**). heart and (**B**). liver.

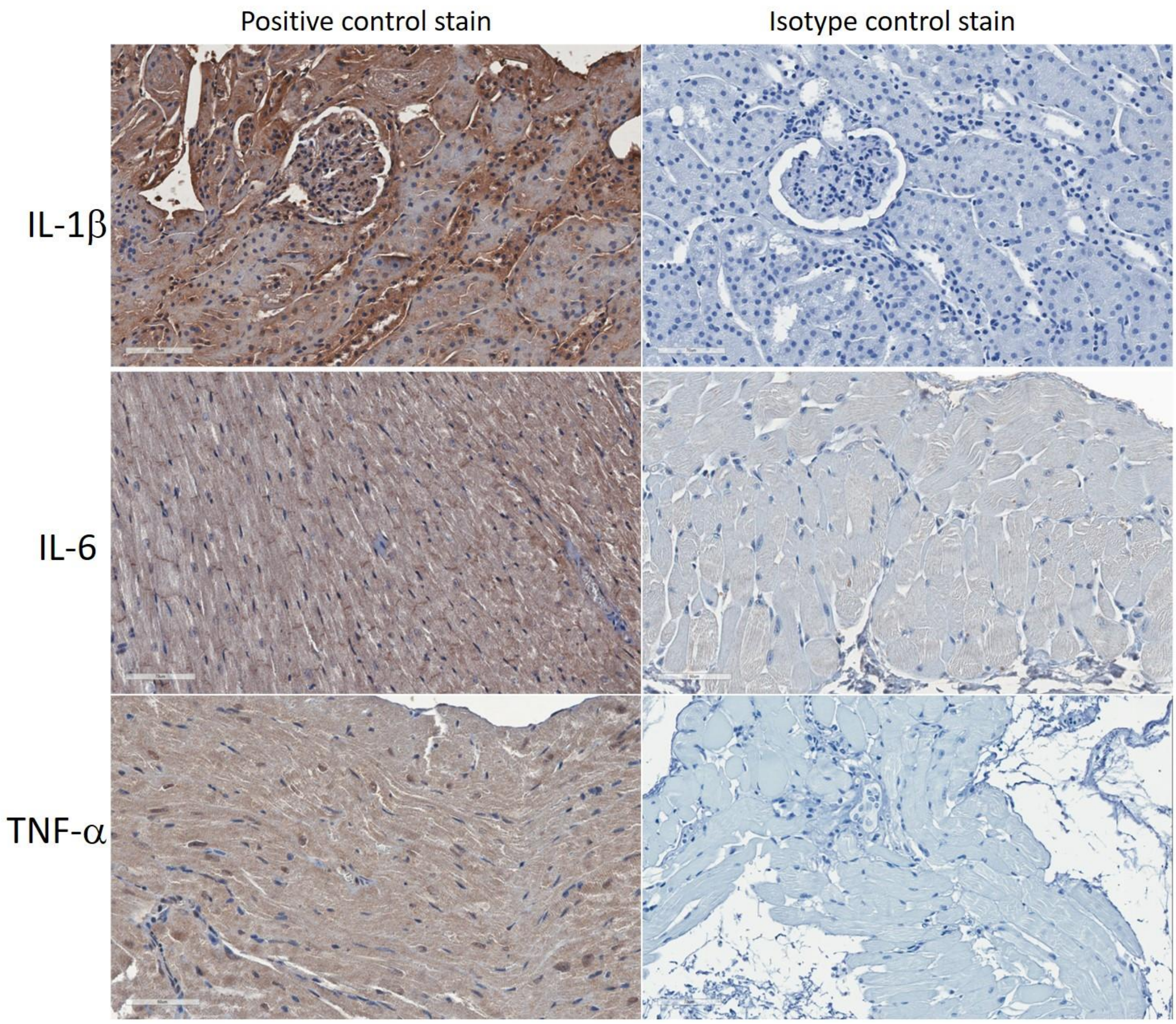

Supplementary Figure 4. Expression of pro-inflammatory cytokines as positive control on tissues other than lung. IL-1β, IL-6 and TNF was stained on kidney and heart tissues respectively as positive control

for their expression. IgG was used as isotype control and served to determine the background and negative stain.

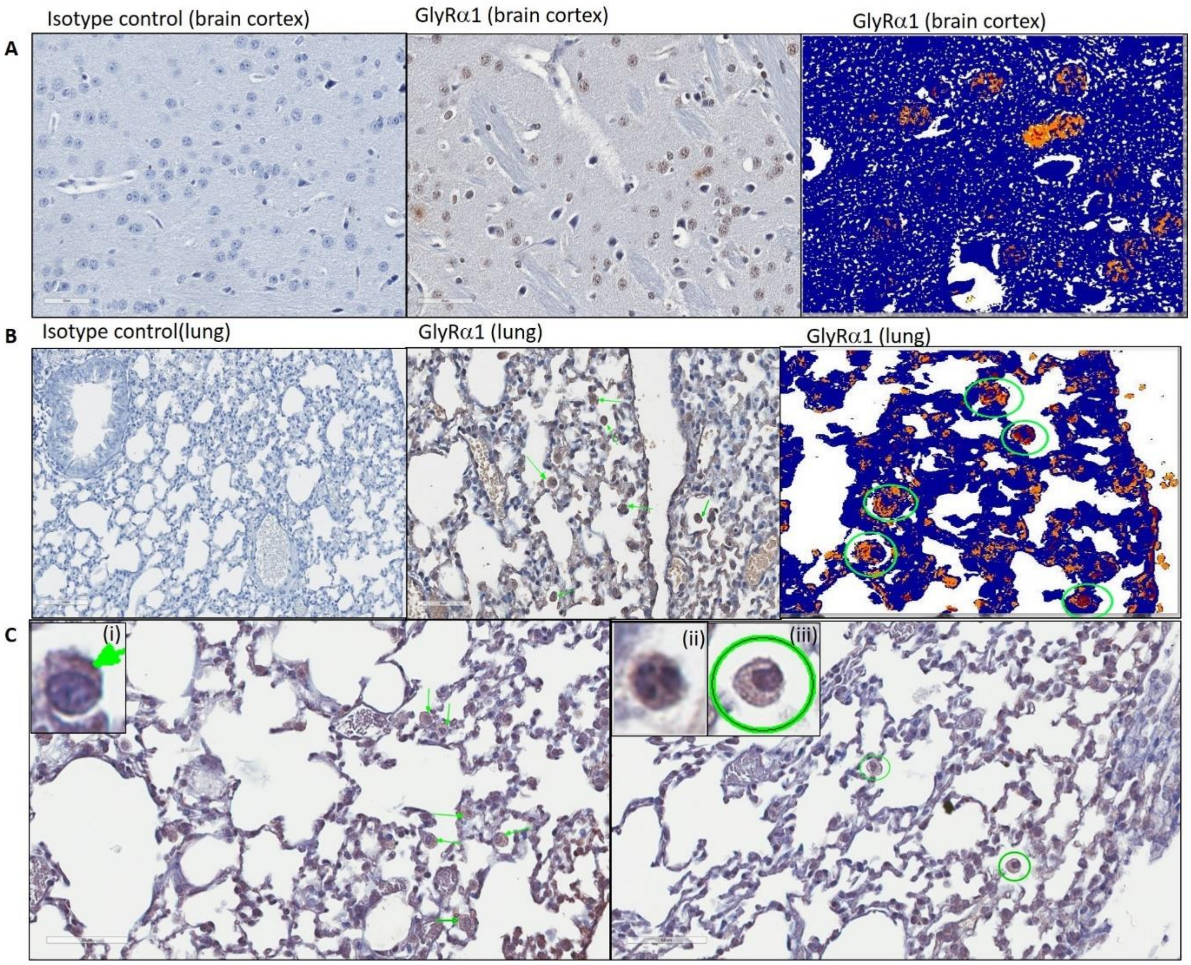

Supplementary Figure 5. Glycine receptor (GlyR) expression on lung immune cells. GlyR expression on paraffin embedded Balb/c mice lung sections was evaluated. (**A**). For positive control, Balb/c mice brain cortex was stained with GlyR-◎1 antibody and at a similar concentration the isotype was used for staining. By positive pixel algorithm, GlyR expression true stain was identified on brain cortex. (**B**). GlyR expression on lung sections at the same concentration as brain cortex. The arrows and ovals point to different immune cells stained in the process. Aperio positive pixel count algorithm was used to identify true stain which showed different immune cells circled in green. (**C**). Specific staining of different immune cells was identified on the lung sections on the basis of the cell shape and nuclei. (**i**) Lymphocyte, (**ii**) Neutrophil and (**iii**) Alveolar macrophage. Many alveolar macrophages were visible and was confirmed by positive pixel count demonstrated in B.
